# Impact of multimodal intervention on cognitive function and nutritional status in older adults with oral frailty: Post‐hoc sub‐group analyses of the J‐MINT study

**DOI:** 10.1002/alz.088654

**Published:** 2025-01-09

**Authors:** Junya Nakamura, Kazuaki Uchida, Taiki Sugimoto, Koichiro Matsuo, Takashi Sakurai, Hidenori Arai

**Affiliations:** ^1^ National Center for Geriatrics and Gerontology, Obu, Aichi Japan; ^2^ University of Washington, Seattle, WA USA; ^3^ Tokyo Medical and Dental University, Tokyo Japan

## Abstract

**Background:**

In Japan, effective interventions to prevent low nutrition in older people with oral frailty have not been well established. This post‐hoc sub‐group analysis of the Japan‐multimodal intervention trial for the prevention of dementia (J‐MINT) aimed to examine the efficacy of the multidomain intervention in older adults with and without oral frailty.

**Methods:**

J‐MINT was an 18‐month randomized controlled trial in which participants aged 65‐85 years with mild cognitive impairment were randomized to a multimodal intervention group (physical exercise, nutrition counseling, cognitive training, and vascular risk factor management) and a control group. This study included the participants in J‐MINT who had undergone the intervention program and follow‐up evaluation at least once. Oral frailty was defined as oral frailty (Oral Frailty Index‐8 ≥ 4 points) at baseline assessment. Outcomes were the change from baseline to 18‐month assessment in cognitive function (the composite scores of eight neuropsychological tests), Body Mass Index (BMI), nutritional status (Mini Nutritional Assessment Short‐Form), fat‐free mass, fat mass, food diversity, and appetite (Council on Nutrition Appetite Questionnaire). A mixed model for the repeated measure was used to calculate the mean difference (MD) of outcome between the intervention and control groups.

**Results:**

Of 531 participants enrolled in J‐MINT, 433 participants were included (mean age: 74.4±4.9; women, 226; intervention group: 215), and 152 participants had oral frailty at baseline assessment. In older adults with oral frailty, although no significant mean difference in cognitive function was observed (MD [95%CI] = 0.099 [‐0.019‐0.217]), significant intervention effect in nutritional status (0.548 [0.034‐1.062]), fat‐free mass (0.842 [0.216‐1.468]), fat mass (‐0.962 [‐1.890–0.038]), food diversity (1.263 [0.374‐2.150]), and appetite (1.315 [0.524‐2.110]) were observed. However, in older adults without oral frailty, there is a significant intervention effect in only food diversity (1.454 [0.808‐2.100]).

**Conclusion:**

The results of this study suggest the effectiveness of multimodal interventions on nutritional indicators for older adults with oral frailty. Oral frailty may be an effective target for multimodal intervention.

